# Hearing and justice: The link between hearing impairment in early childhood and youth offending in Aboriginal children living in remote communities of the Northern Territory, Australia

**DOI:** 10.1186/s40352-019-0097-6

**Published:** 2019-10-30

**Authors:** Vincent Yaofeng He, Jiunn-Yih Su, Steven Guthridge, Catia Malvaso, Damien Howard, Tamika Williams, Amanda Leach

**Affiliations:** 1 Menzies School of Health Research, Charles Darwin University, Casuarina, Northern Territory NT 0811 Australia; 2 University of Adelaide, Adelaide, South Australia SA 5005 Australia; 3 Phoenix Consulting, Nightcliff, Northern Territory NT 0810 Australia

**Keywords:** Hearing impairment, Hearing loss, Youth offending, Delinquency, Aboriginal children, Remote communities, Data-linkage, School attendance, Child maltreatment, Social determinants of health, Social determinants of crime

## Abstract

**Background:**

High prevalence of chronic middle ear disease has persisted in Australian Aboriginal children, and the related hearing impairment (HI) has been implicated in a range of social outcomes. This study investigated the association between HI in early childhood and youth offending.

**Method:**

This was a retrospective cohort study of 1533 Aboriginal children (born between 1996 and 2001) living in remote Northern Territory communities. The study used linked individual-level information from health, education, child protection and youth justice services. The outcome variable was a youth being “found guilty of an offence”. The key explanatory variable, hearing impairment, was based on audiometric assessment. Other variables were: child maltreatment notifications, Year 7 school enrolment by mother, Year 7 school attendance and community ‘fixed- effects’. The Cox proportional hazards model was used to estimate the association between HI and youth offending; and the Royston R^2^ measure to estimate the separate contributions of risk factors to youth offending.

**Results:**

The proportion of hearing loss was high in children with records of offence (boys: 55.6%, girls: 36.7%) and those without (boys: 46.1%; girls: 49.0%). In univariate analysis, a higher risk of offending was found among boys with moderate or worse HI (HR: 1.77 [95% CI: 1.05–2.98]) and mild HI (HR: 1.54 [95% CI:1.06–2.23]). This association was attenuated in multivariable analysis (moderate HI, HR: 1.43 [95% CI:0.78–2.62]; mild HI, HR: 1.37 [95% CI: 0.83–2.26]). No evidence for an association was found in girls. HI contributed 3.2% and 6.5% of variation in offending among boys and girls respectively. Factors contributing greater variance included: community ‘fixed-effects’ (boys: 14.6%, girls: 36.5%), child maltreatment notification (boys: 14.2%, girls: 23.9%) and year 7 school attendance (boys: 7.9%; girls 12.1%). Enrolment by mother explained substantial variation for girls (25.4%) but not boys (0.2%).

**Conclusion:**

There was evidence, in univariate analysis, for an association between HI and youth offending for boys however this association was not evident after controlling for other factors. Our findings highlight a range of risk factors that underpin the pathway to youth-offending, demonstrating the urgent need for interagency collaboration to meet the complex needs of vulnerable children in the Northern Territory.

## Introduction

In the past decade, there has been increasing awareness of the commonalities between the social determinants of criminal behaviour and the social determinants of health, including factors such as poverty, child maltreatment, education and environmental health (Caruso 2017). The description ‘multiple and complex’ has been used to describe the breadth and depth of the needs of vulnerable populations that span a wide range of social and health issues (Rosengard et al. 2007). One significant health issue for people in the criminal justice system (CJS) is hearing impairment (HI). It has been suggested that involvement in the CJS may be a consequence of hearing-related social problems such as low educational standards, unemployment, alcohol and other substance abuse (Howard et al. 1994). Other reports have also suggested that people with HI are more likely to encounter ‘language and learning challenges’, which might lead to ‘challenging behaviours’ (Glickman 2019; Vernon and Greenberg 1999) that increase the risk of CJS engagement. The association between HI and criminal activity was also proposed in a submission to the Inquiry into Hearing Health in Australia (2010), which stated that while HI may not directly cause criminal behaviour, it does have an impact on self-concept, educational attainment and social skills which in turn increase the risk of criminal activity (Australian Government Senate Community Affairs References Committee 2010).

Middle ear disease (otitis media) is a common, usually transient, childhood disease and is the most common cause of hearing loss in children (Australian Institute of Health and Welfare (AIHW) 2014; Kong and Coates 2009; Leach and Morris 2017; Snodgrass and Groves 2017), however for Aboriginal Australian children living in remote communities, it is a major public health issue (Leach 1999; Morris et al. 2007). The prevalence of otitis media among Aboriginal children living in remote communities in the Northern Territory (NT) of Australia is among the highest reported worldwide (Morris et al. 2005), with a recent survey reporting that almost all (90%) children have a history of otitis media (Leach et al. 2016). In this socially disadvantaged population, otitis media tends to affect children early in life, tends to be persistent and is often asymptomatic (Kong and Coates 2009; Lehmann et al. 2008). The prevalence of otitis media has been reported to peak at 5–9 months of age for Aboriginal children in Australia (Lehmann et al. 2008). If left untreated or not treated adequately, otitis media often results in conductive hearing loss, which reduces children’s exposure to language, affects their developmental outcomes and life trajectories. The impact of HI in early childhood on the development of children is supported by recent studies, which have demonstrated that among NT Aboriginal children, those children with HI have an increased risk of lower school readiness at age 5 years, decreased school attendance in Year 1 (at age 6 to 7 years), lower academic achievement in Year 3 (8 to 9 years) and increased contact with the child protection system (Su et al. 2019; Su et al. 2019b).

Studies have also documented a high proportion (94%) of HI among inmates in NT prisons (Vanderpoll and Howard 2011, 2012). However, worldwide, it has been claimed that the CJS is currently ‘ill prepared to accommodate’ people with HI who are ‘both victims and victimizers’ (O’Rourke et al. 2013)—a vulnerable population which has *‘multiple and complex needs*’ (Rosengard et al. 2007) and have often ‘experienced various forms of traumatisation, and oppression’, and are at risk of being ‘further mistreated within the CJS’ (O’Rourke et al. 2013). Previous studies have demonstrated an association between HI and child maltreatment, and between child maltreatment and delinquency, however there have been no large-scale studies that have investigated the link between HI and juvenile delinquency in the Australian Aboriginal population (He et al. 2019b; Malvaso et al. 2016; Malvaso et al. 2017; Su et al. 2019b). These types of studies are important to inform effective prevention and early intervention strategies for juvenile delinquency including in the NT where Aboriginal children comprise about 40% of all NT children (Australian Bureau of Statistics (ABS) 2014, 2016) and are over-represented in both child protection notifications (80%) and in youth detention centres (96%) (AIHW 2017b, 2018).

In the last decade, there has been a growing recognition of the importance of a public health approach to child protection and youth justice to improve interagency collaboration and integrated service delivery (He et al. 2019a; Ombudsman South Australia 2013; Royal Commission and Board of Inquiry into the Protection and Detention of Children in the Northern Territory 2017c). This same decade has also seen the development of data-linkage infrastructure (He et al. 2019a, 2019b; Royal Commission and Board of Inquiry into the Protection and Detention of Children in the Northern Territory 2017b) which has enabled the construction of an NT multi-agency longitudinal research dataset to investigate long-term outcomes, identify predictors of a range of health and social outcomes, and to inform evidence-based interventions (Child Family Community Australia (CFCA) 2011; He et al. 2019a; O'Donnell et al. 2008; Royal Commission and Board of Inquiry into the Protection and Detention of Children in the Northern Territory 2017c). By using linked data from multiple agencies, this study aimed to investigate the association between HI in early childhood and youth offending, to inform interagency collaboration in the provision of effective and culturally responsive early intervention programmes to vulnerable children and families. We believe the findings of this study will have important implications for the recent Australian Government’s ‘Closing the Gap Refresh’ (Council of Australian Governments 2018) and the NT Government’s Reform Implementation Plan (Northern Territory Government 2017, 2018) in response to the Royal Commission into the Protection and Detention of Children in the Northern Territory (Royal Commission and Board of Inquiry into the Protection and Detention of Children in the Northern Territory 2017a, 2017c, 2017d).

## Methods

### Study design and cohort selection

**Fig. 1:**
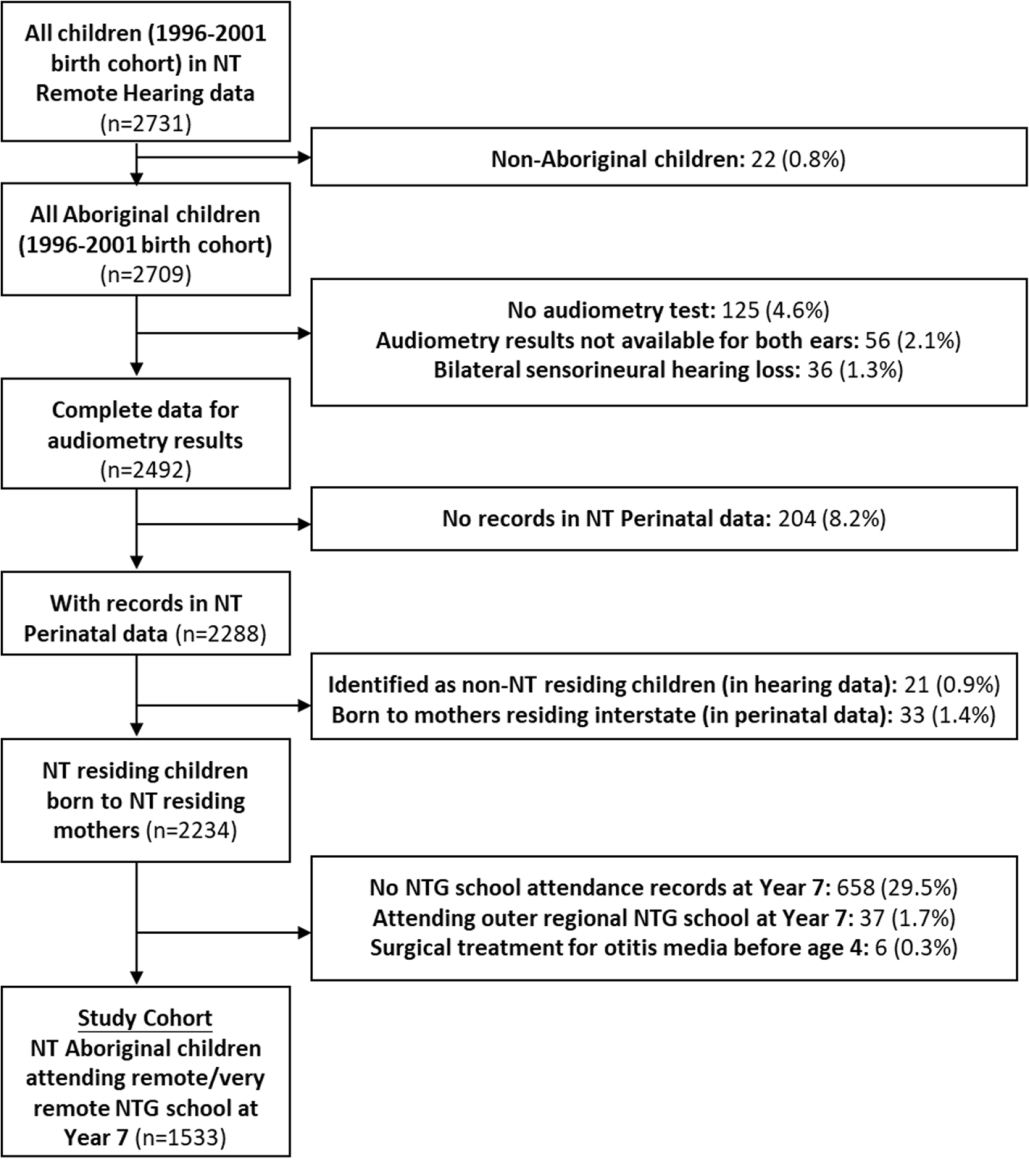
Flow diagram of cohort selection

This is a retrospective cohort study which used linked, de-identified, individual-level administrative data from four government agencies (NT Departments of Health, Education, Territory Families and Attorney-General and Justice). The study cohort was all Aboriginal children, born in the NT between 1st January 1996 and 31st December 2001, with complete audiometric records (for both ears) and who attended an NT Government school in the remote and very remote regions at Year 7. The records for children from the NT Remote Hearing Assessment dataset were linked to NT Perinatal Registry data and NT Government school data to define the study cohort. Hospital inpatient data were used to exclude children with a history of hospital admission for otitis media-related surgery before the age of 4 years, under the premise that early surgery may alter the outcome of HI on child development. The selection process for the study is presented in Fig. 1. After selection, there were 1533 children in the study cohort who completed a hearing assessment between 2007 and 2015.

### Data sources

Data was obtained through the NT child and youth data repository (He et al. 2019b) which had been previously developed as a collaboration between Menzies School of Health Research and NT Government agencies. For this study, six core datasets were used. The **NT Perinatal Data Register** is a statutory register with information recorded for all births in the NT. The **Remote Hearing Assessment** dataset contains clinical information for all children assessed by NT Remote Hearing Services (AIHW 2017a). NT Remote Hearing Services is an outreach service that assesses children referred through the community clinic. Overall it has been reported that 18% of NT Aboriginal people, aged under 21, have received the service (AIHW, 2017a) The **NT Government school dataset** contains public school enrolment and attendance data. The **child protection dataset** includes statutory records of children with any contact with child protection services and includes information on all notifications (reports) of possible maltreatment, substantiated cases and placements in out of home care. All adults are mandated under NT legislation (Section 26(1) of the *Care and Protection of Children Act 2007 [NT]*) to report events in which there is reasonable concern that a child has been harmed or is at risk of harm. Notifications and substantiated cases are classified as one of four types of abuse or neglect – emotional abuse, physical abuse, sexual abuse or neglect. The **NT Hospital Separations Dataset** is a single dataset containing hospital admissions data for all six NT public hospitals. The hospital data was used to identify children who had had surgical procedures related to otitis media. The sixth core dataset was the **NT Integrated Justice Information System** (IJIS) which contains records for individuals charged with an offence and the results of subsequent justice assessments.

### Analysis

#### Outcome variable

The outcome variable was ‘youth offending’ which was defined as the first record of an offence that have been “proven guilty” in the courts. Proven guilty offences were chosen instead of convictions because under the Youth Justice Act 2005 (NT), local court judges have discretion whether or not to formally record a conviction even when charges have been legally proven.

#### Key explanatory variable—hearing impairment

In our study, HI was determined from the first audiometric assessment result, for each child, in the Remote Hearing Assessment dataset under the assumption that the first assessment result was representative of a child’s HI status in their early years. This assumption is supported by previous findings that OM in NT Aboriginal children develops early in life (Lehmann et al. 2008), is persistent and asymptomatic, and is not diagnosed until an older age due to easier diagnosis and greater healthcare access (Morris et al. 2005). In the NT Remote Hearing Service, hearing assessments were performed using pure tone audiometry with results reported as the average threshold of hearing for the three frequencies: 500 hertz (Hz), 1000 Hz and 2000 Hz. The result for each ear was classified as either normal or one of four levels of hearing loss, namely mild (16–30 dB HL), moderate (31–60 dB HL), severe (61–90 dB HL) and profound (≥ 91 dB HL), a comparatively conservative classification which has been deemed more suitable for children aged under 15 (AIHW 2017). Only results of conductive and mixed hearing loss were included in the study. Based on these hearing results, we have derived the HI variable as a categorical variable containing four mutually exclusive categories:

- Normal hearing: normal audiometry results in both ears.
- Unilateral hearing loss (UHL): normal in one ear and any degree of hearing loss in the other.
- Mild HI: mild hearing loss in the better hearing ear.
- Moderate or worse HI: moderate or worse hearing loss in the better hearing ear.

#### Other explanatory variables

In the NT, the minimum legal age of criminal responsibility is 10 years. Therefore, we chose the explanatory variables that were available close to age 10, to identify opportunities for agencies to intervene to prevent first delinquency. These are the proxy variables for the underlying factors of delinquency, in which the links to delinquency have been documented in previous literature (Abrams and Freisthler 2010; Bender 2012; Bernburg and Thorlindsson 2007; He et al. 2019b; Henry et al. 2012; Hirschfield and Gasper 2011; Huang and Ryan 2014; Leiber et al. 2009; Li et al. 2011; Mack et al. 2007; Malvaso et al. 2016, 2017; Wells and Rankin 1991):

1. **Child maltreatment experience** (early and middle childhood) was constructed from the child protection data based on the primary types of child maltreatment notification(s) before entering Year 7 at school. This is an ordinal variable with six mutually exclusive categories: no notification of maltreatment, neglect only, emotional abuse only, physical abuse only, sexual abuse only, and multi-type maltreatment (defined as two or more types of maltreatment). When more than one maltreatment type is recorded for a single event, the primary maltreatment type is the one which is the greatest immediate risk to the child. When a child has been reported for different maltreatment types in more than one child protection notification before Year 7 (e.g. physical abuse at Year 1 and emotional abuse at Year 6), the child was deemed as having multi-type maltreatment before Year 7.
2. **School engagement** was constructed from the school attendance data defined as the proportion of school days attended in Year 7, a categorical variable with three levels: less than 60%, from 60% to 80%, and more than 80% attendance.
3. Whether or not a child was **enrolled by mother** in Year 7 is a dichotomous variable (whether enrolled by mother or enrolled by another person) based on school enrolment records.
4. **Community factors** were included in both the descriptive and regression analysis. The descriptive analysis included two community level factors: the relative remoteness of the community was defined using Accessibility and Remoteness Index of Australia (ARIA+) (Australian Bureau of Statistics (ABS) 2018), and community-level socioeconomic-disadvantage was defined using the Index of Relative Socio-Economic Disadvantage (IRSD) from ABS. In the regression model **Community effects** were incorporated based on the school attendance data for the community in which the child attended Year 7. The location of the community was defined using Australian Standard Geographic Classification (ASGC) for statistical reporting at the level of statistical local area (SLA) (ABS 2007).

#### Aboriginal status

To resolve inconsistency of the recording of Aboriginal status between datasets, the Aboriginal status variable was derived from a hierarchy of accuracy which was based on systematic evaluation of the completeness and quality of each dataset referenced to health records for which an audit, in 2011, demonstrated 98% consistency between recorded Aboriginal status and patient interview (Foley et al. 2012). This approach is described in detail elsewhere (Silburn et al. 2018) and is consistent with best practice guidelines involving data linked from two or more datasets (AIHW and ABS 2012).

#### Statistical analysis

All statistical analyses were conducted using Stata for Windows, Version 15 (StataCorp 2015). Analyses were stratified by sex based on literature suggesting different mechanisms and developmental trajectories leading to offending between males and females (Ferrante 2013a, 2013b; Johansson and Kempf-Leonard 2009; Topitzes et al. 2011).

Survival analysis methods were used to examine the association between HI and a record of youth offence. Survival time was defined as the time in years from the tenth birthday to the occurrence of first “proven guilty” offence (the legal age of criminal responsibility in the NT is 10 years), and children who had not committed first offence were censored at the earliest of the following: date of death, the last observed date in the linked data or the end of the study period on 31 December 2017. The time-scale for the survival analysis was the age of children in years (continuous variable).

Information on last contact with a child recorded in the data repository were used for recording (censoring) whether a child remained in the NT. These datasets include health (primary care consultations, hospital admissions and child immunisations), education, death registration, child protection and juvenile justice data (He et al. 2019a). This step avoids the risk of including children in the analysis who may have migrated out of the NT and for whom there would be incomplete records.

The Kaplan–Meier estimator method was used to estimate the cumulative proportion of first youth offence which describes the proportion of the population which is estimated to have experienced the event over time. (Kaplan and Meier 1958) The cumulative proportion was estimated for each of the four categories of HI.

In the multivariable analysis, the Cox proportional hazard method was used to examine the association between HI and the youth offending (defined by first offence) (Andersen and Gill 1982). Given the characteristics of our study cohort (Aboriginal children living in remote and very remote communities), the targeted outcomes of children from the same community are likely to be correlated. To account for any intra-group (community) correlation, the standard errors were clustered at the community level. To account for cohort effects, a separate baseline hazard rate was estimated for each birth cohort.

The Royston R^2^ measure was used to estimate the separate contribution of each risk factor in the variations in youth offending (Royston 2006). The Royston R^2^ measure has been previously used in a Queensland study (Thomas et al. 2015) to quantify the contribution of health-related factors for re-incarceration and in a Swedish study (Witt et al. 2015) to estimate the proportion of variation (in violent crime of schizophrenia patients) explained by prior criminal history factors. It has also been used to investigate the contribution of various factors in explaining the risk of readmission following surgery in the United States (Merkow et al. 2015).

## Results

Selected baseline characteristics for boys and girls in the study cohort are presented in Table 1. The majority of the study cohort resided in areas that were in the most socioeconomically disadvantaged category by IRSD (boys: 88.9%; girls: 89.2%) and very remote (boys: 89.8%; girls: 90.2%). More than one-third of the cohort had been reported to the child protection system before Year 7 (boys: 37.4%; girls 33.1%). Over 70% of children were recorded as being enrolled by their mother at Year 7 (boys: 70.7%; girls: 76.0%) and more than 40% recorded an attendance rate of < 60% in Year 7 (boys: 44.6%; girls: 43.4%).

**Table 1:** Characteristics of cohort (number and proportion (%))

Characteristic	Boys (*n* = 754)	Girls (*n* = 779)
Audiometry results		
Normal hearing	393 (52.1%)	403 (51.7%)
Unilateral hearing loss	154 (20.4%)	170 (21.8%)
Mild hearing impairment	158 (21.0%)	143 (18.4%)
Moderate or worse hearing impairment	49 (6.5%)	63 (8.1%)
Age of first audiometry test (median)	10.2	10.2
Contact with child protection system before Year 7^a^ (mutually inclusive group)		
Any child maltreatment notification	282 (37.4%)	258 (33.1%)
Any neglect notification	169 (22.4%)	141 (18.1%)
Any physical abuse notification	103 (13.7%)	92 (11.8%)
Any sexual abuse notification	65 (8.6%)	72 (9.2%)
Any emotional abuse notification	57 (7.6%)	60 (7.7%)
Any out-of-home care placement	33 (4.4%)	26 (3.3%)
Child maltreatment notification before Year 7^a^ (mutually exclusive group)		
No child maltreatment report	472 (62.6%)	521 (66.9%)
Reported for neglect only	98 (13.0%)	78 (10.0%)
Reported for physical abuse only	51 (6.8%)	40 (5.1%)
Reported for sexual abuse only	26 (3.4%)	36 (4.6%)
Reported for emotional abuse only	19 (2.5%)	23 (3.0%)
Reported for more than one abuse type	88 (11.7%)	81 (10.4%)
Educational experience at Year 7		
Enrolled by mother at Year 7	533 (70.7%)	592 (76.0%)
School attendance at Year 7		
Less than 60%	336 (44.6%)	338 (43.4%)
> =60% and less than 80%	234 (31.0%)	253 (32.5%)
> =80%	184 (24.4%)	188 (24.1%)
Community level factors		
Living in most disadvantaged areas^b^	670 (88.9%)	695 (89.2%)
Living in very remote regions	677 (89.8%)	703 (90.2%)

^a^Child protection notifications that occurred after the first youth offending were excluded to maintain the correct temporal order between the child maltreatment and youth offending and to avoid ambiguity that might arise when youth crime precedes maltreatment

^b^Defined as being in the lowest quintile of Index of Relative Socio-Economic Disadvantage (IRSD) based on community where the children go to school at Year 7

The median age of the first audiometry test was 10.2 years in both boys and girls. Almost half of the study cohort had UHL/HI (boys: 47.9%: girls 48.3%). Among the children in our study cohort with no history of an offence (boys: 612; girls: 730), 46.1% of boys and 49.0% of girls had UHL/HI. Among the children with a record of being found guilty of an offence (boys: 142; girls: 49), 55.6% of boys and 36.7% of girls had UHL/HI.

### Cumulative proportion of first proven guilty offending

**Fig. 2:**
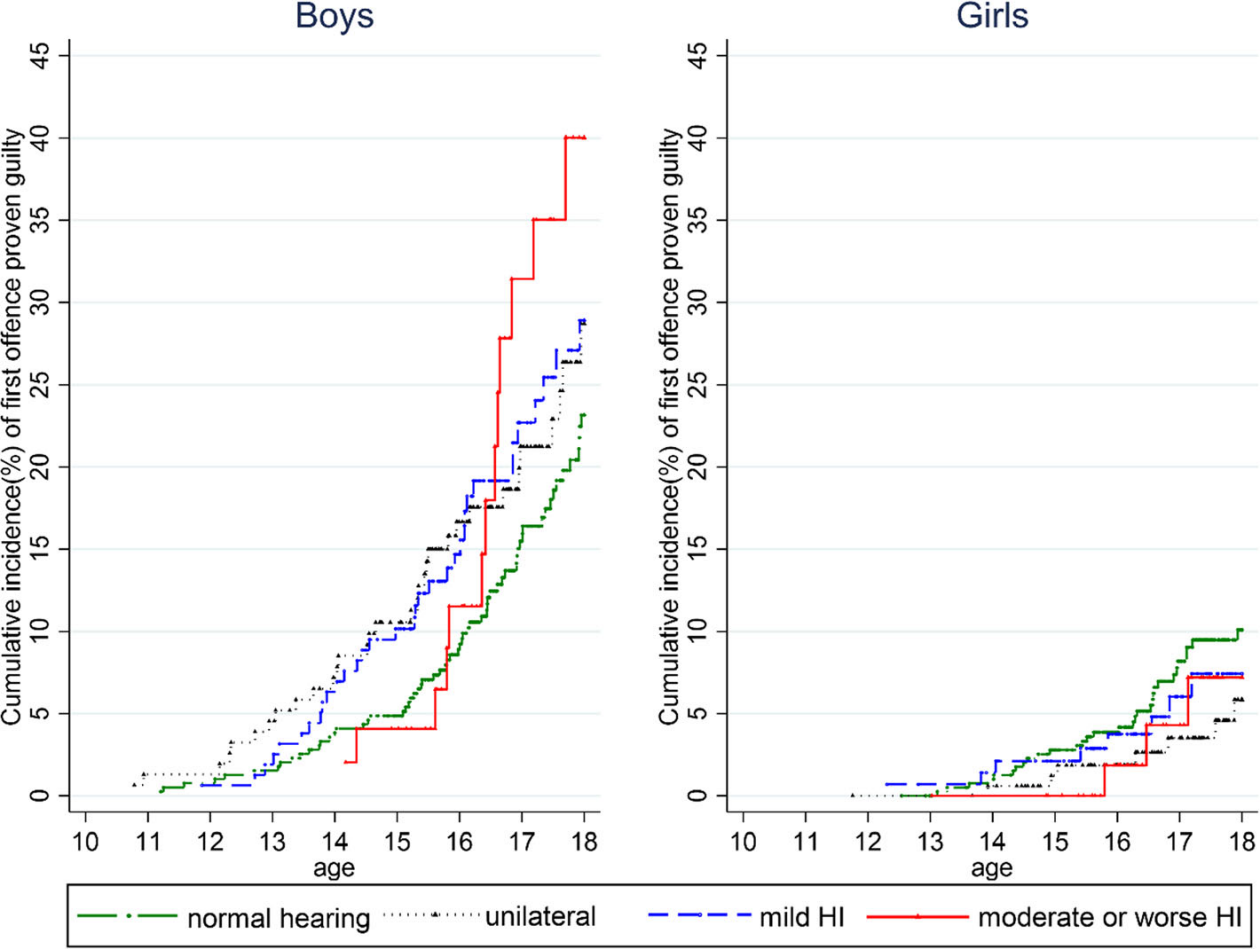
Survival analysis depicting the cumulative proportion of first proven guilty offending (from 1 Jan 2006 to 31 Dec 2017) at different ages, by sex and different levels of hearing impairments (HI) for NT-born Aboriginal children in NT remote hearing data and NTG school attendance (Year 7) data (1996–2001 birth cohort)

Figure 2 shows the Kaplan-Meier failure curves of first proven guilty offence for both boys and girls with varied levels of HI. For each level of HI, boys had a higher risk of offending than girls. The univariate analysis revealed the difference in the association between HI and offending for boys and girls. For boys, those with moderate (or worse) hearing loss had the highest cumulative proportion of youth offending (40.0%; 95% CI: 24.8–60.1%), followed by boys with mild (28.9%; 95% CI: 20.9–39.1), UHL (28.7%; 95% CI: 20.3–39.6), and normal hearing (23.2%; 95% CI:18.2–29.2). For girls, those with normal hearing had the highest cumulative proportion of youth offending (10.1%; 95% CI: 7.1–14.2), followed by girls with mild (7.4%; 95% CI: 3.7–4.7), moderate (or worse) (7.2%; 95% CI:2.3–21.1) and UHL (5.9%; 95% CI:2.7–12.2).

### Factors associated with offending (including HI)

The risk of offending was significantly higher in boys with a record of moderate or worse HI (HR: 1.77, 95% CI:1.05–2.98, *p* = 0.031) and those with mild HI (HR: 1.54, 95% CI:1.06–2.23, *p* = 0.023) than in boys with normal hearing in the univariate analysis (Table 2). These associations were attenuated when child maltreatment, school factors (school attendance and enrolment by mother), and community fixed-effects were added to the multivariable model, with no evidence for an association between HI and offending (moderate HI, HR: 1.43, 95% CI = 0.78–2.62, *p* = 0.252; mild HI, HR: 1.37, 95% CI = 0.83–2.26, *p* = 0.215).

**Table 2:** Univariate and multivariable¶ analysis for youth offending (first offence proven guilty), NT-Aboriginal children in (1996–2001 NT birth cohort) in NT remote hearing data and public school attendance data (Year 7)

	Boys				Girls			
	Univariate Analysis		Multivariable Analysis		Univariate Analysis		Multivariable Analysis	
Characteristic	HR(95% CI)	*p*	HR(95% CI)	*p*	HR(95% CI)	*p*	HR(95% CI)	*p*
Hearing impairment (HI)								
Normal hearing^a^	1.00		1.00		1.00		1.00	
Unilateral hearing loss	1.43 (0.99–2.06)	0.058	1.32 (0.78–2.23)	0.303	0.50 (0.18–1.37)	0.177	0.45 (0.15–1.32)	0.145
Mild HI	1.54 (1.06–2.23)*	0.023	1.37 (0.83–2.26)	0.215	0.70 (0.35–1.39)	0.304	0.68 (0.34–1.37)	0.281
Moderate or worse HI	1.77 (1.05–2.98)*	0.031	1.43 (0.78–2.62)	0.252	0.57 (0.27–1.18)	0.13	0.66 (0.27–1.63)	0.37
Child maltreatment notification before Year 7								
No child maltreatment report^a^	1.00		1.00		1.00		1.00	
Only reported for neglect	2.11 (1.46–3.03)***	< 0.001	1.69 (1.17–2.46)**	0.006	2.28 (1.21–4.30)*	0.011	1.08 (0.43–2.73)	0.869
Only reported for physical abuse	2.28 (1.38–3.77)**	0.001	2.24 (1.33–3.79)**	0.002	4.01 (1.83–8.81)***	0.001	4.33 (2.30–8.14)***	< 0.001
Only reported for sexual abuse	2.38 (1.07–5.29)*	0.034	1.92 (0.82–4.48)	0.134	0.62 (0.12–3.21)	0.571	0.54 (0.08–3.82)	0.538
Only reported for emotional abuse	0.96 (0.31–2.98)	0.946	0.65 (0.21–2.02)	0.46	1.20 (0.14–10.06)	0.866	1.30 (0.15–11.12)	0.809
Reported for more than one abuse type	3.23 (1.82–5.71)***	< 0.001	2.71 (1.54–4.79)***	0.001	4.67 (2.62–8.32)***	< 0.001	4.49 (2.03–9.94)***	< 0.001
Enrolled by mother at Year 7								
No^a^	1.00		1.00		1.00		1.00	
Yes	0.90 (0.59–1.37)	0.623	1.20 (0.78–1.84)	0.407	0.28 (0.20–0.40)***	< 0.001	0.38 (0.28–0.52)***	< 0.001
School attendance at Year 7								
Less than 60%^a^	1.00		1.00		1.00		1.00	
> =60% and less than 80%	0.65 (0.51–0.83)***	0.001	0.67 (0.52–0.86)**	0.002	0.75 (0.43–1.30)	0.300	1.05 (0.54–2.04)	0.889
> =80%	0.44 (0.30–0.66)***	< 0.001	0.49 (0.33–0.73)***	0.001	0.26 (0.07–1.06)	0.061	0.33 (0.08–1.32)	0.117

* *p* < 0.05; ** *p* < 0.01; *** *p* < 0.001; ^a^ Reference categories. ^¶^ Adjusted for community ‘fixed effect’

In the multivariable model for boys (Table 2), risk of youth offending was found to be significantly higher for children reported for more than one type of maltreatment (HR: 2.71, 95% CI: 1.54–4.79; *p* = 0.001) before Year 7, followed by physical abuse only (HR: 2.24, 95% CI: 1.33–3.79; *p* = 0.002), and neglect only (HR: 1.69, 95% CI: 1.17–2.46; *p* = 0.006). Risk of youth offending was found to be significantly lower for boys with Year 7 attendance between 60% and 80% (HR: 0.67, 95% CI: 0.52–0.86; p = 0.002) and above 80% (HR: 0.49, 95% CI: 0.33–0.73; p = 0.001) than boys with Year 7 attendance less than 60%.

For girls, the association between HI and offending was not evident in either univariate nor multivariable analysis (Table 2). In the multivariable analysis, the risk of offending was higher for girls with multi-type maltreatment (HR: 4.49, 95% CI: 2.03–9.94; *p* < 0.001) and physical abuse only (HR: 4.33, 95% CI: 2.30–8.14; p < 0.001). There was also strong evidence for a lower risk of offending for girls who were enrolled by mothers at Year 7 (HR: 0.38, 95% CI: 0.28–0.52; *p* < 0.001).

*Contribution of individual categories in explaining the variations in offending (fixed effect model).*

In the ‘fixed effect model’, the contribution of explanatory variables factors in explaining the variations in offending was quantified using the Royston R^2^ measure (Table 3). The multivariable model explained a greater proportion of the variation in offending for the girls (58.5%) than boys (28.1%). HI contributed 6.5% of the variation in offending among girls but 3.2% for boys. At the individual level, the variation in youth offending was partially explained by child maltreatment notification before Year 7 (boys: 14.2%; girls 23.9%) and Year 7 school attendance (boys: 7.9%; girls: 12.1%). Enrolment by mother at Year 7 also explained some of the variation in offending for girls (25.4%), but not for boys (0.2%). ‘Community fixed effects’ accounted for the greatest proportion of explained variance for both boys (14.6%) and girls (36.5%).

**Table 3:** Contribution of individual categories in explaining the variation (Royston R^2^) of first youth offending guilty in the ‘fixed-effect’ model for NT-born Aboriginal children (1996–2001 birth cohort) in NT remote hearing data and public school attendance data (Year 7)

Model	Royston R^2^	
	Boys (*n* = 754)	Girls (*n* = 779)
Full model	0.281	0.585
Hearing impairment only	0.032	0.065
Maltreatment report before Year 7 only	0.142	0.239
Enrolled by mother (at Year 7)	0.002	0.254
Year 7 school attendance only	0.079	0.121
Community fixed effect only	0.146	0.365

## Discussion

This study is the first to use multiple linked administrative datasets to examine the association between audiometrically-diagnosed conductive HI and youth-offending among Australian Aboriginal children. The study has also quantified the relative contribution of HI and other markers of social wellbeing and engagement as predictors of youth-offending. Our analysis found that the pattern and magnitude of association differ between boys and girls. For boys, both ‘moderate or worse HI’ and ‘mild HI’ were associated with higher risk of offending in the univariate analysis, although in both categories, the association was attenuated in the multivariable model. No evidence for an association was found in either the univariate, or the multivariable model for girls. The reasons for the lack of an association for boys after adjustment for other factors is unclear, however it is possible that any impact of HI was masked by the much stronger impact caused by the experience of child maltreatment and community factors.

### High rates of HI among Aboriginal children in the justice system

Our study demonstrates that the prevalence of HI among Aboriginal children with a record of an offence is high, which corroborates the results of a previous study on HI among adult inmates in Darwin and Alice Springs prisons (Vanderpoll and Howard 2011, 2012). As previously described, the high rates of hearing problems among NT Aboriginal prisoners raises concern about their ability to communicate with corrections staff, a situation that can be exacerbated for those for whom English is not their first language (Vanderpoll and Howard 2011). Given that Aboriginal youth make up 96% of all youth justice detainees in the NT and that the NT has the highest prisoner recidivism rate (58.3%) in Australia (Northern Territory Government Department of the Attorney-General and Justice 2017), addressing the high rates of HI in Aboriginal children and improving the response of the justice system to hearing-impaired Aboriginal people may be a critical step in reducing Aboriginal incarceration rates (Vanderpoll and Howard 2011). These findings support the need for additional support for young detainees and prisoners with HI, including routine hearing assessment for new detainees, better access to hearing aids, sharing results of detainee’s past hearing assessments by the health department with police and the courts, and improved training for police, the judiciary and correctional staff in identifying the signs of HI among individuals and ways to improve communication with them (Vanderpoll and Howard 2011).

### Child maltreatment and youth offending

Our study also reaffirms findings from previous studies of an association between exposure to child maltreatment and youth offending (Ferrante 2013a, 2013b; He et al. 2019b; Hurren Paterson 2015; Hurren Paterson et al. 2017; Malvaso 2017; Malvaso et al. 2016, 2017; Royal Commission and Board of Inquiry into the Protection and Detention of Children in the Northern Territory 2017a; Stewart et al. 2008). Our results provide new insight into the separate association between child maltreatment notification and youth offending for boys and girls in the study cohort. Second to community factors, child maltreatment notification explained the largest proportion of variation in youth offending, for both boys and girls. The highest risk of youth offending was found in children reported for more than one type of maltreatment. This finding corroborates the results of a previous study, which found that a high proportion of Aboriginal children recorded in both the NT child protection and youth justice system had been reported for all four types of maltreatment (He et al. 2019b). This result supports the development of targeted intervention programs for children who have contact with child protection, particularly those who have been notified for multiple types of maltreatment, in order to prevent later contact with the youth justice system.

### School attendance and youth offending

Our study provides encouraging evidence that increased school attendance in Year 7 is associated with a lower risk of offending, particularly for the boys. This result supports efforts to maintain the engagement of students to prevent later contact with the justice system. More than two-fifths of the study cohort had a school attendance (Year 7) rate of less than 60%. This finding is consistent with previous research that reported a decline in school attendance from Year 6 onwards, with average attendance rates for Aboriginal children living in very remote regions dropping from almost 70% in Year 5 to less than 60% in Year 7 (He et al. 2018). In this context, promoting and maintaining student engagement in school is particularly important during the middle years, and is consistent with previous studies that have reported the mediating effects of school engagement in the relationship between maltreatment and delinquency (Bender 2012; Malvaso et al. 2016).

### Community factors and youth offending

Our study found that community factors explained the greatest proportion of the variation in youth offending in remote communities. These findings emphasise the importance of “interventions that focus not only on the child’s offending behaviour, but also on key aspects of a child’s social environment” in addressing the complex and multiple needs of children and youth in the NT (Royal Commission and Board of Inquiry into the Protection and Detention of Children in the Northern Territory 2017a). The result reinforces the need for place-based strategies for community safety and crime prevention, and the importance of partnerships between government and local communities that are “built on the principles of mutual respect, shared commitment, shared responsibility and good faith” (Royal Commission and Board of Inquiry into the Protection and Detention of Children in the Northern Territory 2017c). However, research that further elucidates the associations between communication problems related to hearing loss, community level factors, child maltreatment and youth offending is needed to inform prevention and intervention strategies to reduce youth crime in the NT (Abrams and Freisthler 2010; Coulton et al. 2007; Freisthler 2004; Freisthler et al. 2007; Huang and Ryan 2014). Such research could promote the interagency collaboration and coordination of children and family services needed to better support vulnerable families (Hovmand et al. 2007; Royal Commission and Board of Inquiry into the Protection and Detention of Children in the Northern Territory 2017a).

### Limitations

There are several limitations to the study. Firstly, not all Aboriginal children living in remote areas accessed the ear health outreach service and not all attended government schools. Care must be taken with generalisation of the results to all NT Aboriginal children or all Aboriginal children living in remote communities. The new ‘Hearing for Learning’ program is expected to increase the proportion of young NT Aboriginal children receiving regular ear and hearing assessment with the potential for more representative data (Menzies School of Health Research 2019). A second limitation is that the availability and timing of the hearing assessment (AIHW 2017a) made it necessary to use each child’s first audiometry result for analysis, under the assumption that the result was indicative of long-term HI status of a child, as most of the children in our study cohort had their first audiometric assessment at middle childhood (around the age of 10 years). The prevalence of chronic middle ear disease peaks at early years, then declines through childhood. There may be children whose development was affected by HI at an earlier age, but who later have improved hearing. These cases will result in misclassification leading to an underestimation of the strength of association between a record of HI and youth offending. A third limitation is that the outcome measure of “proven guilty” is a precise but narrow measure of youth offending. As other datasets, such as police data, become available there is opportunity to extend the research on hearing impairment to other measures of offending, including apprehension by police and referral to youth diversion programs. With more comprehensive linked data on youth offending there is also opportunity for analysis of other dimensions of offending, such as the age of first offending and the frequency of offending. A final limitation is that there are other factors such as child and parental hospital admissions (relating to mental health, substance use and assault) that may be important confounders but which were not available for inclusion in the multivariable models.

## Conclusion

Our study confirms a high prevalence of HI in remote Aboriginal children with and without offending records. There was evidence, in univariate analysis, for an association between HI and youth offending, for boys only, however this association was not evident after controlling for other risk factors. Our findings highlight the complex range of factors that underpin youth offending for remote Aboriginal children including the relatively greater impacts of child maltreatment and community factors on youth offending than HI, and the different risks for youth offending between boys and girls. These findings point to opportunities for early intervention to disrupt the pathway into the youth justice system, and provide a clear message for governments, policy makers, and community service providers about the urgent need for interagency collaboration to meet the ‘multiple and complex needs’ of vulnerable children in the Northern Territory (Rosengard et al. 2007; Royal Commission and Board of Inquiry into the Protection and Detention of Children in the Northern Territory 2017a; Vidal et al. 2019).

## Data Availability

The study datasets contain sensitive personal information and are held on a secure cloud-based server with restricted access. Access requires the approval of the ethics committee and data custodians.
